# Resolving the Heterogeneous Tumor-Centric Cellular Neighborhood through Multiplexed, Spatial Paracrine Interactions in the Setting of Immune Checkpoint Blockade

**DOI:** 10.1158/2767-9764.CRC-21-0146

**Published:** 2022-02-10

**Authors:** Rachel L.G. Maus, Alexey A. Leontovich, Raymond M. Moore, Laura Becher, Wendy K. Nevala, Thomas J. Flotte, Ruifeng Guo, Jill M. Schimke, Betty A. Dicke, Yiyi Yan, Svetomir N. Markovic

**Affiliations:** 1 Department of Oncology, Mayo Clinic, Rochester, Minnesota.; 2 Department of Immunology, Mayo Clinic, Rochester, Minnesota.; 3 Department of Biomedical Statistics and Informatics, Mayo Clinic, Rochester, Minnesota.; 4 Department of Anatomic Pathology, Mayo Clinic, Rochester, Minnesota.

## Abstract

Direct interactions between tumor and immune cells mediate the antitumor effect of all modern cancer immunotherapeutic agents. Simultaneously, tumor cells have evolved mechanisms of evasion, including the downregulation of HLA-I, potentially disrupting the mechanism of action employed by many immune checkpoint inhibitors. And yet, the *in situ* interplay between these cells within the tumor immune microenvironment (TIME) remains elusive. Recent advances in histologic multiplex bioimaging platforms have enabled in-depth molecular characterization of single cells within spatially preserved and clinically archived tumor tissues. Herein, we applied multiplex immunofluorescence to excisional lymph node biopsies from 14 patients with metastatic melanoma who experienced clear objective responses to immunotherapy (7 complete response; 7 progressive disease) to determine distinguishing features of the TIME in the pretreatment setting. Distinct regions of the TIME were evaluated using 35 proteins probing tumor, immune, and vasculature components across 323 fields of view. Single-cell compositional analysis confirmed established prognostic immune cell types including increased prevalence of cytotoxic T cells within the tumor core fields of view of responders. Integrating single-cell quantification with the spatial arrangement of cellular neighborhoods surrounding tumor cells revealed novel, spatial immune signatures capable of stratifying TIME based on clinical response. Our analysis revealed dynamic cellular composition of the tumor-centric cellular neighborhood (TCCN) based on anatomic subregion, functional expression of HLA-I by the index tumor cell and ultimately clinical response to immunotherapy. Overall, this study provides an analytic framework to resolve the cellular complexity of the TIME, increasingly relevant to the outcomes of modern cancer immunotherapy.

**Significance:**

Findings from this work propose a novel approach to resolving clinical heterogeneity of the TIME by objectively quantifying the cellular interactions occurring in metastatic melanoma lymph node tissue utilizing multiplex immunofluorescence. This study provides an analytic and biologically derived unit of measure, the TCCN which is customizable for studying critical paracrine interactions within spatially preserved tissue of various cancers and across the spectrum of multiplex imaging modalities.

## Introduction

Treating metastatic cancer with immunotherapy has dramatically altered the clinical landscape of medical oncology, especially for patients with metastatic melanoma. And yet, clinical responses to immune checkpoint inhibitors (ICI) remain suboptimal and challenging to predict (1–3). Specifically, monotherapy treatment with anti-PD1 has clinical response rates in approximately 44% of patients while combination immunotherapy strategies improve these rates to around 52%, with long-term benefit only in the 15%–30% range (4–6). The underlying mechanisms responsible for these variable clinical responses are multi-faceted and difficult to resolve given heterogeneity at the patient, tissue, cellular, and molecular levels. Beyond tumor intrinsic factors, an increasing role for the surrounding tumor microenvironment is becoming realized as a major source of interpatient and intrapatient heterogeneity.

The influence of the tumor immune microenvironment (TIME) on cancer progression, metastasis, and therapeutic responsiveness has been studied by numerous groups and is now recognized as a pillar in the hallmarks of cancer (7, 8). Historically, isolated features of the TIME have been studied resulting in an array of critical events which together have the potential to create a tumor-permissive microenvironment in various tissue types. These features span from tumor-centric mechanisms of immune evasion including downregulation of HLA-I on the surface of tumor cells (9, 10) to active recruitment of immune suppressive cells such as regulatory T cells and M2-polarized macrophages to promote tumor survival and proliferation in the microenvironment (11). Collectively, these studies highlight the dynamic nature whereby bidirectional cross-talk between tumor and immune cells gives rise to a coordinated TIME.

For metastatic melanoma, and most solid tumor malignancies, the lymph node is the most common, first site of metastatic dissemination (12, 13). Serving as the epicenter of host immunity, the lymph node is an immunologic organ uniquely equipped and structured to facilitate coordinated immune responses. Under homeostatic conditions, functional subunits of B cell–enriched follicles surrounded by dendritic cells, macrophages, and T cells facilitate direct cell–cell interactions required for antigen presentation and adaptive immune responses (14). In contrast, in the metastatic setting much of this architecture is replaced, resulting in a new landscape of cellular interactions among metastatic tumor cells and resident and recruited immune cell subsets (15, 16). ICI specifically target these cellular interactions to disrupt immune checkpoint mechanisms and reengage the host's antitumor immune response (17). Understanding how the paracrine microenvironment surrounding individual tumor cells relates to variable clinical responses within spatially preserved metastatic tissue may provide a yet unrealized framework for studying the complexity of the heterogeneous TIME.

To deconstruct the inherent complexity of the TIME, bioimaging modalities have rapidly evolved to enable deep profiling at single-cell resolution. These technologies were first applied to liquid biopsies and single-cell suspensions but have more recently been developed for formalin-fixed paraffin-embedded tissue (18). Currently, a suite of single-cell, multiplex bioimaging platforms have emerged utilizing mass spectrometry and cyclic immunofluorescence–based methodologies capable of quantifying the phenotypic, functional status and spatial position of individual cells within a preserved tissue landscape (19). To date, these technologies have been largely applied to tissue microarrays to comprehensively assess the TIME across distinct tissue regions among patients (20–24). As such, there remains a critical need for applying these technologies to preserved, whole tissue sections utilizing robust, versatile spatial frameworks to provide biologically and clinically relevant units of measure.

In this study, we applied a cyclic multiplex immunofluorescence (MxIF) platform to archival lymph node tissue to characterize the TIME of metastatic melanoma in 14 treatment-naïve patients with clear objective responses to anti-PD1 therapy. Utilizing a 35-analyte panel, the MxIF platform enabled single-cell, quantitative evaluation of tumor and immune cell subsets within microanatomic regions of the metastatic lymph node including the tumor core, tumor margin, and lymphoid tissue. To interrogate paracrine interactions of tumor cells derived from these distinct regions, we developed a biologically derived spatial metric, the tumor-centric cellular neighborhood (TCCN). This spatial metric was applied to the metastatic lymph node to evaluate how differences within the tumor-immune paracrine landscape relate to distinct anatomic regions, molecular features of individual tumor cells and clinical outcomes in patients undergoing ICI therapy.

## Materials and Methods

### Study Population

Thirty treatment-naïve patients with metastatic melanoma who received anti-PD1 immunotherapy as part of their treatment course were selected for MxIF analysis in the current study. From this initial cohort, 14 cases with clear objective responses as defined by RECIST, complete response (responders) versus progression (nonresponders) to immunotherapy were selected for subsequent analysis. Analogous to the clinical response rates reported to anti-PD1 treatment in stage IV melanoma (4–6), our study identified 7 patients (23%) had a complete response to immunotherapy while an additional 7 patients (23%) experienced progressive disease following treatment. Median follow-up for patients was 34 months. Patient demographics and treatment course are summarized in Table 1. Use of all patient biospecimens and clinical data were collected in accordance with the Declaration of Helsinki following approval by Mayo Clinic's Institutional Review Board (IRB). Given the retrospective, minimal-risk structure of the study design, informed written consent from participants was waived by the IRB. Research authorization was verified for each patient prior to use of samples for research purposes.

**TABLE 1:** Patient summary demographics.

	Progressive disease (nonresponders) (*n* = 7)	Complete response (responders) (*n* = 7)
**Sex (male/female)**	7/0	5/2
**Median age at time of biopsy (range)**	60 (41–83)	53 (51–74)
**Stage at time of biopsy**		
* Stage III*	7	6
* Stage IV*	0	1
**Site of surgical excisional biopsy**		
* Lymph node*	7	7
**Days from diagnosis to ICI treatment**	239 (12–657)	204 (11–734)
**ICI therapy**		
* Single (anti-PD1)*	4	4
* Combination (anti-PD1 with anti-CTLA4 or anti-IDO)*	3	3
**Median time to follow-up (months)**	9.4 (1.1–29.6)	49.9 (25.5–80.5)

### Antibody Panel Selection

The panel was designed with 35 antibodies capable of elucidating the phenotypic and functional status of the tumor, immune, vascular, and structural components of the lymph node TIME. A summary of all antibodies is provided in Supplementary Table S1. The validation of MxIF antibodies was evaluated by an experienced board-certified dermatopathologist who handles melanoma pathology on a daily basis. In selected antibodies, the performance of the MxIF stains was compared with that of routine chromogenic IHC stains to ensure complete concordance between these two methods. For the antibodies without concurrent IHC stains, the corresponding hematoxylin and eosin (H&E) sections were reviewed to recognize different cell types in the sections, including tumor cells, lymphocytes, histiocytes, endothelial cells, and fibroblasts, and to determine the cell distributions; the MxIF stains were subsequently evaluated to ensure their match to the appropriate cell type and distribution based on the corresponding H&E features (Supplementary Table S1). Only the antibodies meeting the above validation criteria were chosen for our MxIF study, which also underwent subsequent random quality checking to ensure the repeatability, as well as revalidation when there was an antibody lot change.

After image acquisition the antibodies were evaluated again for overall performance in the study-specific tissue including staining specificity, signal-to-noise ratio, autofluorescence (AF) removal, and artifact detection. As a result, three antibodies were removed from the final analysis. Antibody panel design enabled phenotypic and functional differentiation of tumor and immune cell subsets as detailed in our classification pipeline.

### Antibody Purification and Conjugation

Rabbit and mouse anti-human antibodies were first purified on HiTrap Protein A or Protein G columns respectively to remove additives and stabilizers. Buffer exchange and concentration was conducted using Amicon 30K filters and purified antibody was resuspended in PBS buffer at a final concentration of 1 mg/mL. Direct conjugation to Cy2-, Cy3-, and Cy5-Bis NHS Ester dye fluorophores (GE Healthcare PA12000, PA13000, PA15000) was done according to manufacturer's instructions. The conjugation reaction was prepared with a minimum loading of 35 μg of protein, 10% w/v of NaHCO_3_ (pH 8.5), and dye conjugate and incubated for 60 minutes in the dark at room temperature. Following the conjugation, the reaction was loaded on a Zeba desalt spin column (Thermo Fisher Scientific) to remove unbound fluorophore, and the final concentration and dye:protein ratio measured by Nanodrop. Following conjugation, the antibody solution was adjusted to a final concentration between 150 and 300 μg/mL and stabilized with 1% BSA and 0.45% sodium azide.

### Slide Preparation

Slide preparation and antigen retrieval was done as described previously (21). Briefly, a 5 μm tissue section affixed to a positively charged glass slide was baked for 60 minutes at 60°C, deparaffinized in xylene and rehydrated in a graded series of alcohol (ethanol:deionized water, 100:0, 95:5, 70:30, 50:50; 10 minutes each). Slides were washed twice in PBS and 0.3% Triton X-100 prior to antigen retrieval. A two-step heat-induced epitope retrieval was conducted in NxGen decloaking chamber (Biocare Medical) using citrate buffer (pH 6, Vector Labs) for 20 minutes heated to 110°C followed by an additional 20 minutes in heated Tris-EDTA buffer (pH 8.5). Following cooling, the slides were washed in PBS (four washes; 5 minutes each) then blocked with 10% donkey serum in PBS for 60 minutes. Slides were washed in PBS, stained with DAPI (1 μg/mL) for 20 minutes followed by a final PBS wash prior to coverslipping with a non-hardening mounting media (90% glycerol, 4% propyl gallate, and 1% DABCO).

### CellDIVE Background Imaging and Fields of View Selection

Images were acquired using the INCell Analyzer 2500HS (GE Healthcare). Following initial DAPI staining, imaging at a 10× objective at all 798 positions across the glass slide area were stitched to provide a whole slide image. A single region of interest including the entire tissue specimen was then selected and a virtual H&E image was created with the DAPI and Cy3 channels. Using RhedEye software, a clinical pathologist annotated the metastatic lymph node tissue to enable selection of fields of view (FOV) that satisfy three anatomic regions of interest: tumor core, tumor margin, and lymphoid tissue (Supplementary Fig. S1). Each FOV maintained the same defined size of 2,040 × 2,040 pixels (∼1 mm^2^). Following FOV selection at the 10× objective, background imaging at the 20× objective was conducted for each FOV at each fluorescence channel (DAPI, Cy2, Cy3, Cy5).

### Multiplex Staining and Dye Inactivation

Staining with multiple antibodies was achieved by alternating rounds of dye inactivation imaging with stained tissue imaging. For immunofluorescence staining, slides were incubated with an antibody cocktail (200 μL) diluted in PBS + 3% BSA (to a final concentration of each antibody between 2.5 and 15 μg/mL) for 1 hour at room temperature in a humidified chamber. Following three PBS washes, the slides were coverslipped for imaging (see Multiplex Imaging Acquisition and Preprocessing section below). Following imaging, dye inactivation was performed by incubating slides in 0.5 mol/L NaHCO_3_, pH 11.2 solution for two washes of 15 minutes each. Following the inactivation, the slides were washed three times in PBS, briefly incubated with DAPI before a final PBS wash and then coverslipped as described previously. Imaging of the dye inactivation round was acquired between each stained round as described below. After image acquisition of a stained or dye inactivation round, the coverslip was removed by inverting slides in a PBS bath with gentle agitation.

### Multiplex Imaging Acquisition and Preprocessing

Images were acquired using the INCell Analyzer 2500HS scanner (GE Healthcare) according to manufacturer's protocol (21). Briefly, background images were acquired before the application of fluorescent-conjugated antibodies using the 20× objective at each annotated FOV on a given slide. Following each stain and dye inactivation round, the FOVs were subsequently imaged at each of the fluorescent channels.

Image files created from acquisition include a raw image, registered image, bleach image, and autofluorescence (AF) removed image. Per-pixel image subtraction of the dye inactivation image from the stained image in the corresponding round was performed for AF removal. After the digital removal of AF, image normalization was applied, which scaled the intensities according to the minimum exposure time in the dataset and then image subtraction was computed per pixel between the stain to dye inactivation captures. This step includes the algorithm accounting for dark pixel intensity offset (minimum intensity value from the microscope; ref. 25). Image alignment was performed for each round using the initial 10× image of DAPI stain. After image capture, image alignment across subsequent stains for every FOV was performed using Insight Toolkit registration, phase correlation on the DAPI channels in each round.

### Pixel Classification–Based Segmentation

To draw distinct, reliable boundaries around each cell and its respective subcellular compartments, we adapted the pixel classification process developed previously by the Bodenmiller lab (22). The separate image files from the INCell were joined into an OME.TIFF file format in order that the respective biomarkers could be visualized simultaneously via other software. This process relies on encoding individual pixels into one of three classes to differentiate nuclear, cytoplasm, and background areas using the Ilastik package, resulting in three probability prediction matrices (26). We enhanced our pixel classification model by using a subset of 14 common core biomarkers (DAPI, CD14, CD163, CD16, CD206, CD20, CD45, CD4, CD68, CD8, gp100, HLA-II, HLA-I, NaKATPase, S6) to increase the number and diversity of training images, while still allowing for overall panel design changes. Pixel classifier training conducted on the common core markers targeted different microenvironment architectures including tumor, lymphatic immune, and vascular cell features. Model training was conducted by labeling randomized crops (200 × 200 pixels) selected from FOVs in the dataset. The single pixel uncertainty was calculated and evaluated on labeled and unlabeled crops to determine the ability for algorithmic detection to distinguish between classes. From this effort, we obtained 11.85% ± 3.9% uncertainty in the 83 training crops, and 11.30% ± 3.6% uncertainty in 256 validation (non-annotated) crops. This model was then applied to every FOV in the dataset for pixel classification and read into CellProfiler for constrained object propagation and the generation of labeled masks for whole cell and nuclei (27). These segmentation masks were merged with the full panel OME.TIFF MxIF image files and incorporated into QuPath, an open-source software package for quantitative bioimaging analysis (28). Using a QuPath script to merge disparate data files, QuPath was then used to export all quantifications from the imported segmentations to analysis R notebooks

### Classification and Single-Cell Analysis

Cell classification was subdivided into two approaches for phenotypic and functional classification, respectively. In phenotypic classification, mutually exclusive cellular subsets were manually assigned to cell clusters using a dimension reduction approach. To better leverage the t-distributed stochastic neighbor embedding (t-SNE) dimension reduction, FOVs were grouped by pathologist assigned label (core, margin, lymphoid tissue; ref. 29). Feature selection for the t-SNE dimension reduction was comprised of the phenotypic markers in the panel design (Supplementary Fig. S2), spatial metrics (area, length, circularity, solidity max and min diameter for nucleus and cell components and the nucleus to cell area ratio), and constitutive markers (DAPI, S6, NaKATPase) capable of differentiating target cell phenotypes. A decision tree with the associated markers can be found in Supplementary Fig. S1. The resulting dimensional reductions t1 and t2 were collated to generate unsupervised clusters via KNN and Louvian community detection (30). Cluster optimization was performed by assessing various k-parameters. The resulting clusters were then manually assigned into phenotypes by trained immunologists. If there was disagreement or ambiguity in a given cluster, it was assigned to the unclassified field and removed from downstream neighborhood analysis. The percent of classified cells was calculated to be greater than 90% in all samples assessed and is detailed in Supplementary Table S2.

For functional classification of a single marker HLA-I expression on tumor cells, a self-organizing map neural network approach through CytoMap was applied to each FOV and across all samples. By this unsupervised approach, manual assessment revealed that a three-classifier model provided optimal sensitivity and specificity of HLA-I expression and cells were assigned to one of three distinct HLA-I classes: high, moderate, or none. For analysis, the classes of high and moderate were combined into HLA-I positive and compared with HLA-I negative (none class; ref. 31). The model was generated using the following input features: the HLA-I fluorescent intensity metrics of median nucleus, median membrane, mean, median, and SD of whole cell and morphology metrics including cellular and nuclear solidity, circularity, length, and area.

Classification labels for both cellular phenotypes and functional expression of HLA-I were returned to QuPath and merged with the spatial coordinates of each cell, its segmentation profile and classification label to visualize and quantify single metrics in all downstream analytics. This per-cell assignment was then mapped back to the FOV and compared with the original MxIF overlay for visual assessment and validation. Abundance plots were used to visualize the immune, tumor and vasculature components present in each FOV. Each cell class distribution was assessed using the Spearman correlation statistic as a nonparametric association between responders and nonresponders. A *P* value of < 0.05 refutes a common association between patient groups, although low counts with significance may be less reliable.

### Definition of TCCN

Analysis of TIME was performed using SpatStat package in R programming language, using cell neighborhood analysis (CNA) algorithm (32). This algorithm uses SpatStat function to traverse through every point on a two-dimensional plane and create a neighborhood of a requested size (50 μm diameter). CNA then counts cells of every class in each neighborhood and records coordinates and counts in a matrix. In the current study, neighborhoods were created around each tumor cell, named TCCN. Derivation of the size of the TCCN for this study is described in Fig. 4. Each TCCN distribution was assessed using the Spearman correlation statistic as a nonparametric association between responders and nonresponders. A value of *P* < 0.05 refutes a common association between patient groups, although low counts with significance may be less reliable.

### Data Availability Statement

The data generated in this study are available within the article and its Supplementary Data files. Supporting data generated in this study are available upon request from the corresponding author.

## Results

**FIGURE 1:**
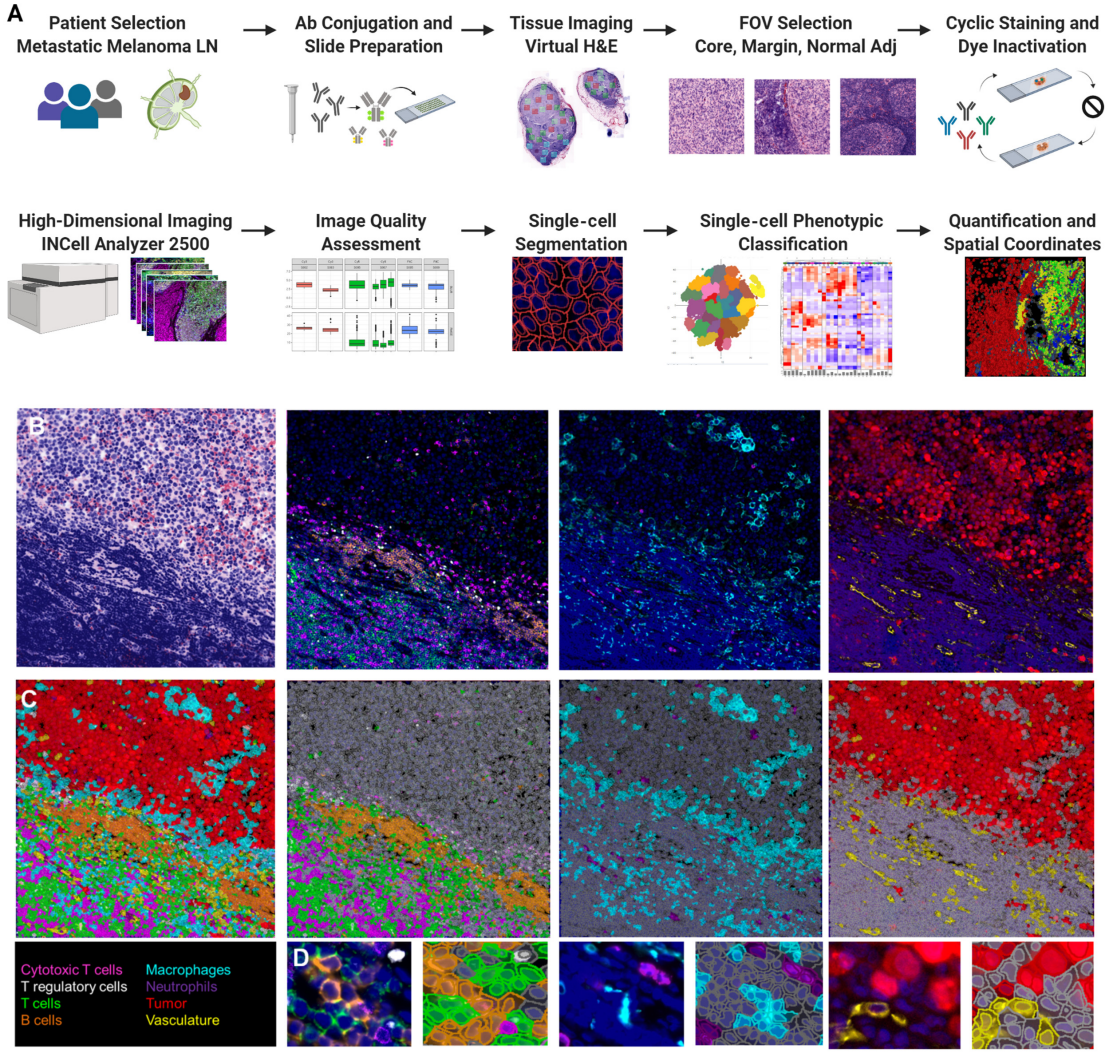
MxIF study design and classification validation. Overview of MxIF workflow (**A**). Visual assessment of classification in QuPath (**B–D**). Virtual H&E of a tumor margin FOV and MxIF images visualizing the lymphocyte, myeloid, tumor, and vasculature components (**B**). Classification mask overlay for the composite image and cell classes (**C**). Inset images to show single-cell classification assignments.

Increasingly, dynamics of the TIME have been recognized for their contribution to variable clinical responses to immunotherapy in the metastatic setting. To quantitatively profile the complexity of the TIME in spatially resolved metastatic tissue, we adapted a cyclic MxIF imaging approach to evaluate surgical excisional lymph node biopsies obtained from treatment-naïve patients with metastatic melanoma who underwent subsequent immunotherapy (Fig. 1A). Through optimization of directly conjugated antibodies, a 35-analyte panel was designed to profile the diversity of the TIME including melanoma-associated antigens, differentiation, and functional markers of immune cell subsets, and structural features including lymphatic and blood vasculature (Supplementary Table S1). From an initial cohort of 30 patients, 14 patients were selected on the basis of clear objective responses (complete response vs. progression) to anti-PD1 therapy (Table 1). From the excisional lymph node biopsy, board-certified pathologists annotated 323 FOVs encompassing distinct anatomic regions of the tumor core, tumor margin, and lymphoid tissue within the excised lymph node. The MxIF imaging and analysis workflow was applied utilizing the 35 antibody panel customized for metastatic melanoma lymph node tissue. A summary of the FOV type selected and corresponding cells segmented and classified are summarized in Supplementary Table S2 including segmentation of over 2 million cells and phenotypic classification achieved in over 90% of cells. Following image acquisition and quality assessment, single-cell segmentation and classification was performed to provide per-cell assignments to tumor, immune, vasculature, or structural phenotypes (Fig. 1B–D). Single-cell quantification of eight major immune cell subsets (B cells, helper T (Th) cells, cytotoxic T cells, regulatory T cells, plasma cells, monocytes, macrophages, and neutrophils), tumor cells, and vasculature components were evaluated for every FOV across all samples.

**FIGURE 2:**
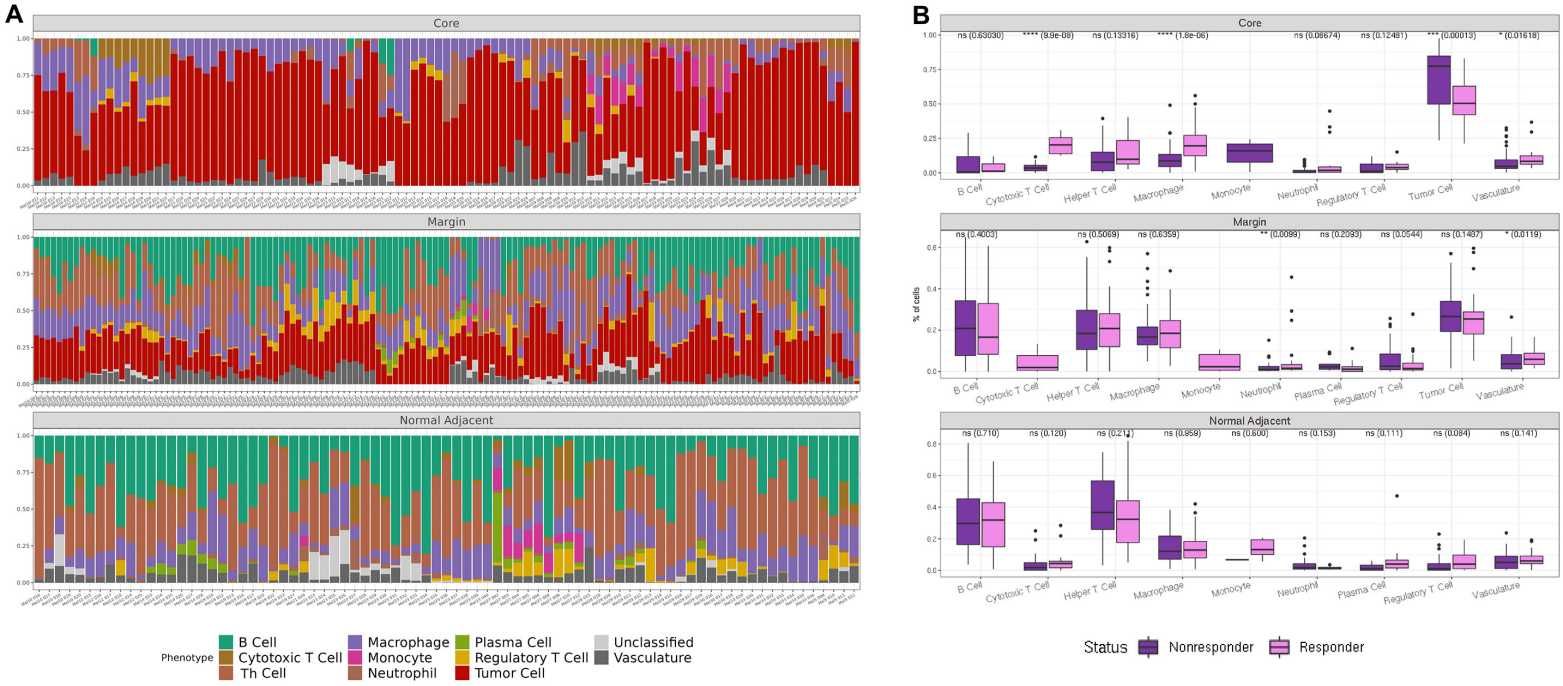
Single-cell composition of metastatic melanoma lymph node by anatomic subregion. Abundance plots of classified cells across all FOVs in tumor core, tumor margin, and normal adjacent lymphoid tissue (**A**). Statistical significance in abundances between cell types in responders and nonresponders (NR; *n* = 136 R FOVs, *n* = 193 NR FOVs; **B**).

Abundance plots summarize the cell counts and proportions of each cell class at the FOV level across the distinct regions of tumor core, margin, and lymphoid tissue. Expectedly, abundance plots reflected the anatomic region sampled with tumor core FOVs displaying the highest tumor class abundancies, lymphoid tissue FOVs displaying primarily immune cell subsets, and interface FOVs comprised of a mixture of all classes (Fig. 2A). Next, single-cell distributions between responder and nonresponders were compared by anatomic region to determine whether inherent differences were present between the two patient cohorts at the single-cell level. The tumor core demonstrated the greatest difference with cytotoxic T cells and macrophages being significantly increased in responders (Fig. 2B).

**FIGURE 3:**
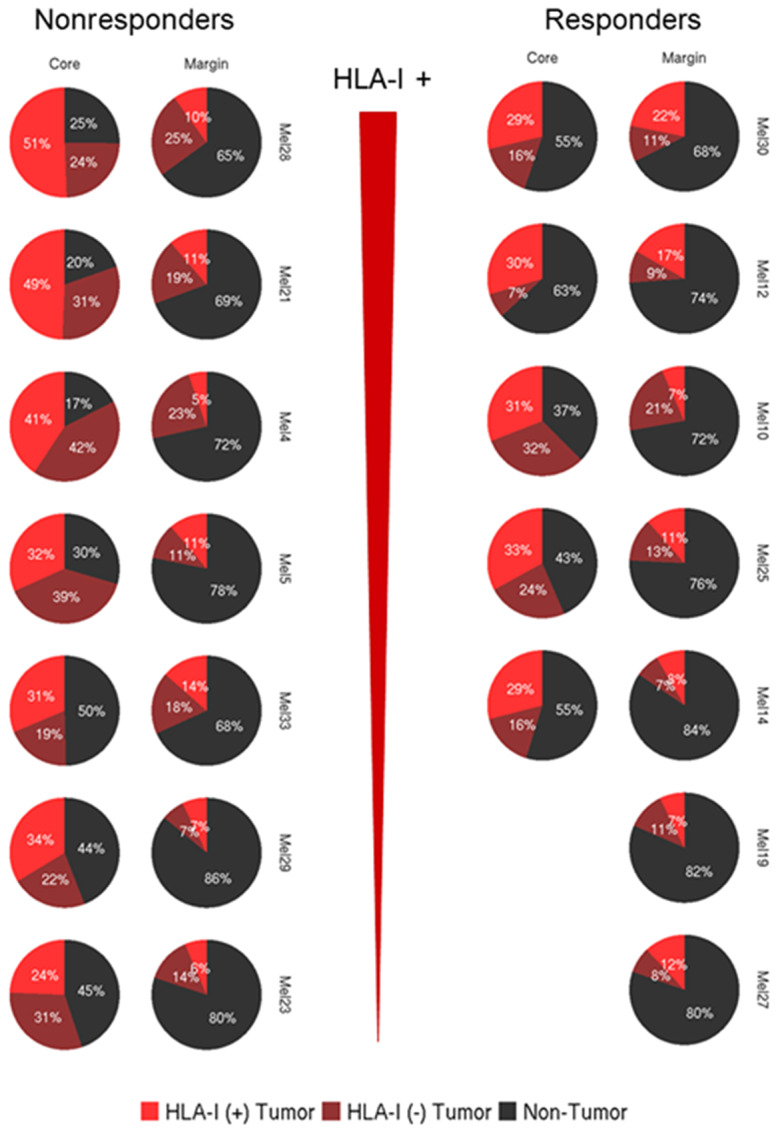
Single-cell expression of HLA-I on classified tumor cells. Proportion of classified tumor cells expressing HLA-I (bright red) versus no expression of HLA-I (dark red).

An established mechanism of immune evasion employed by tumor cells with potential implications in poor response to ICI, is the downregulation of HLA-I expression on the tumor cell surface (10). While a variety of genetic, epigenetic, and microenvironmental mechanisms have been identified as contributing to this range of HLA-I expression on tumor cells, the clinical significance of this variation as it relates to immunotherapeutic response is only beginning to be explored (9, 10). Therefore, we next considered expression of the functional marker HLA-I on tumor cells as a potential discriminator between patients and clinical responses to immunotherapy. By an unsupervised, neural network self-organizing map approach, HLA-I expression level was classified as positive or negative for every cell. Merging phenotypic and functional classification, cells were subdivided into tumor and nontumor components for each of the samples across all FOVs of the core and margin. The tumor cell component was further characterized based on HLA-I expression and ordered according to HLA-I+ tumor cell percentage in responders and nonresponders (Fig. 3). Interestingly, all samples and anatomic subregions had a distribution of tumor cells with and without detectable HLA-I expression on the tumor cell surface; however, there were no significant differences in frequency of HLA-I expression profile that correlated with clinical outcome. Despite the established role of HLA-I in antigen presentation and its critical role in relaying ICI mechanisms of action (9), single-cell resolution of HLA-I was not sufficient to resolve distinct clinical responses to ICI treatment at the patient level.

**FIGURE 4:**
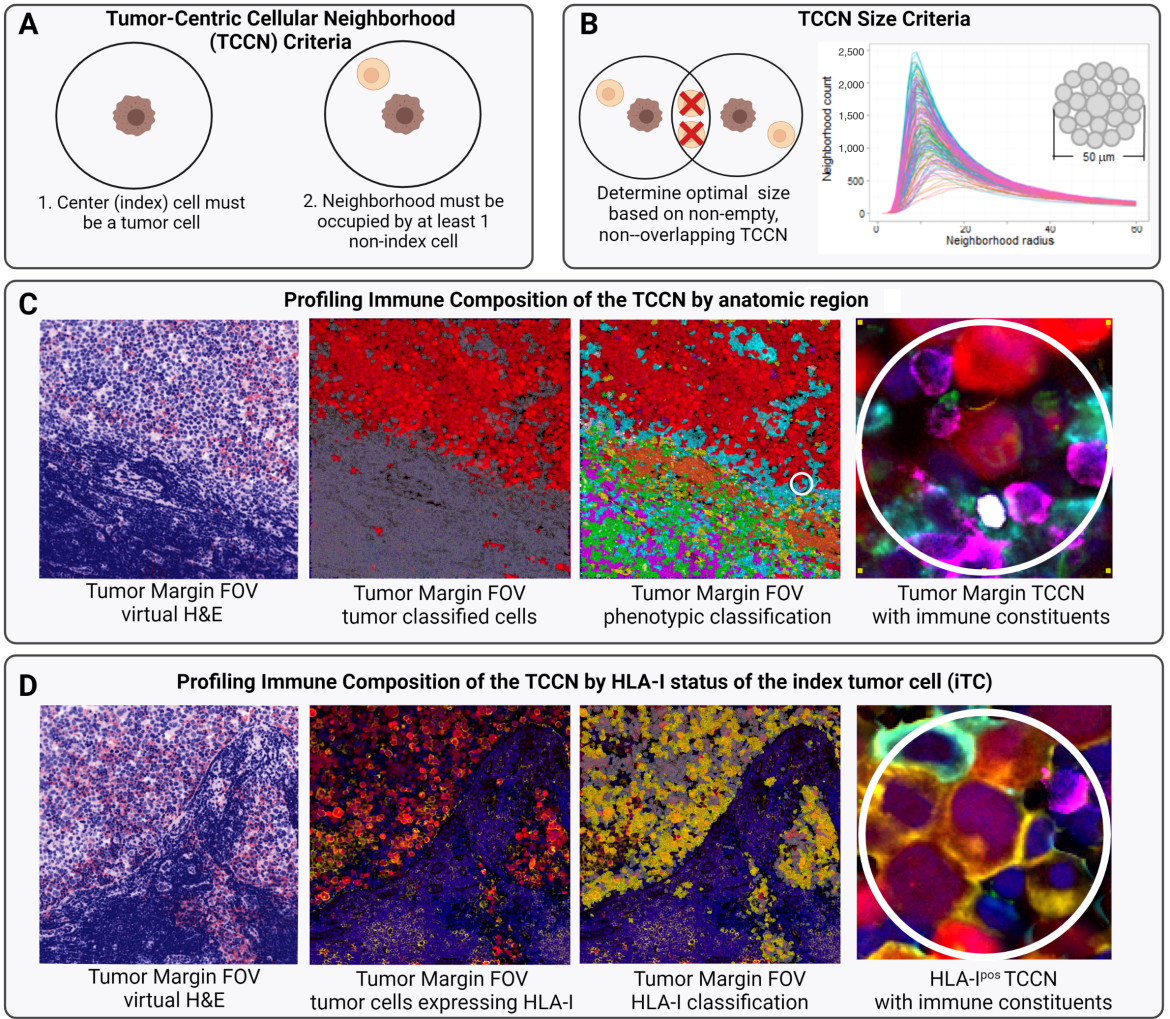
Cellular criteria required for TCCN definition (**A**). Derivation of study-specific 50-μm-diameter TCCN size based on number of neighborhoods satisfying cell and nonoverlapping criteria across various radius sizes (0–60 μm; **B**). Stepwise derivation of TCCN from tumor margin FOV (**C**) and derivation of TCCN based on HLA-I status of the iTC (**D**). Color legend for **C** and **D**: tumor (red), macrophage (teal), B cells (orange), Th cells (green), cytotoxic T cells (pink), regulatory T cells (white), neutrophils (purple). In **D**, HLA-I expression is indicated in yellow. Figure created with Biorender.com.

Building from single-cell quantification at the resolution of anatomic subregions and functional status of tumor cells, we next considered the higher dimensional spatial arrangements of the individual cells and their immediately adjacent cell neighbors to further differentiate distinct TIME. To evaluate the paracrine network of interactions of cells within close proximity to each other, we adapted the method of CNA by modeling a micro-niche surrounding each tumor cell in a given FOV, termed a tumor-centric cellular neighborhood (TCCN). Neighborhood-based analysis was dependent on the following two criteria: (i) the center (index) cell must be classified as a tumor cell and (ii) the neighborhood must be occupied by at least one additional cell beyond the index cell (Fig. 4A). By these two criteria, the optimal TCCN size was computed for the study by considering across all FOVs, the number of TCCNs that satisfy both criteria across increasing radius sizes from 1 to 60 μm (Fig. 4B). For the current tissue and cell type, over 90% of TCCN satisfied both criteria at a radius between 12 and 25 μm. To accommodate cells of varying sizes and particularly myeloid cells with distinct morphologies and extensions, we selected the TCCN size for this study to be 25 μm radius and 50 μm diameter. The derivation of each TCCN in a given FOV can be visualized by overlaying the MxIF image with the tumor class mask and demarking 50 μm diameter regions around each tumor cell (Fig. 4C).

**FIGURE 5:**
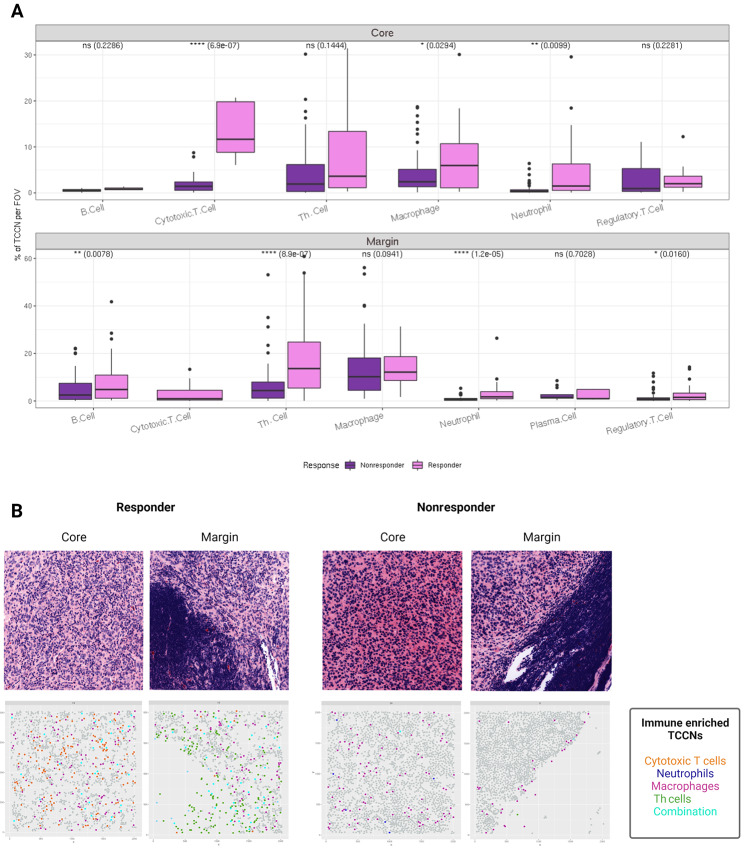
TCCNs by anatomic subregion. Box plots of immune cell subsets populating TCCNs in the tumor core and tumor margin. Comparisons between responder and nonresponder TCCNs were compared using the Spearman correlation statistic (**A**). Virtual H&E and corresponding spatial plots visualizing TCCNs on the FOV colored on the basis of select immune cell subsets or a combination of immune cells in TCCNs of the tumor core and margin representing a responder and nonresponder (**B**). Figure created with Biorender.com.

Utilizing TCCN as the spatial metric of the TIME landscape, we next sought to evaluate the differences in immune composition in TCCNs derived from distinct anatomical subregions of the lymph node: the tumor core and margin. The percent of TCCNs populated by different immune cell phenotypes was determined for tumor core and tumor margin in responders and nonresponders and revealed that overall TCCNs derived from responders had an increased percentage of TCCNs populated with immune cells compared with nonresponder counterparts. Specifically, in core-derived TCCNs, responders had a significant increase in cytotoxic T cells, macrophages, and neutrophil-populated TCCNs while margin-derived TCCNs showed an increase in B cells, Th cells, regulatory T cells, and neutrophil-populated TCCNs in responders (Fig. 5A). Overall, the TCCN spatial analysis resolved additional immune cell counterparts contributing to differences in the TIME between responders and nonresponders beyond single-cell distributions alone.

Mapping this observation within the spatial landscape, TCCNs were plotted back to the anatomical FOV of origin to consider the dominant immune cell type within each TCCN of the tumor core and margin. Considering only the immune cell subsets that distinguished responders from nonresponders in the core and margin respectively, spatial FOVs were visualized for responders and nonresponders. Treating each TCCN as a point on the FOV, the spatial distribution of TCCN in the tumor core versus tumor margin can be visualized. Compared with the corresponding virtual H&E image, TCCNs were more evenly dispersed throughout tumor core FOVs while the spatial localization of TCCNs to the tumor and immune-interfacing edge is apparent in the tumor margin FOV. Notably, the responder TCCNs illustrated more frequent integration among immune cell types within the same TCCN, while the nonresponder TCCN suggests primarily macrophage occupied TCCN comprise the tumor margin at the interacting edge (Fig. 5B).

**FIGURE 6:**
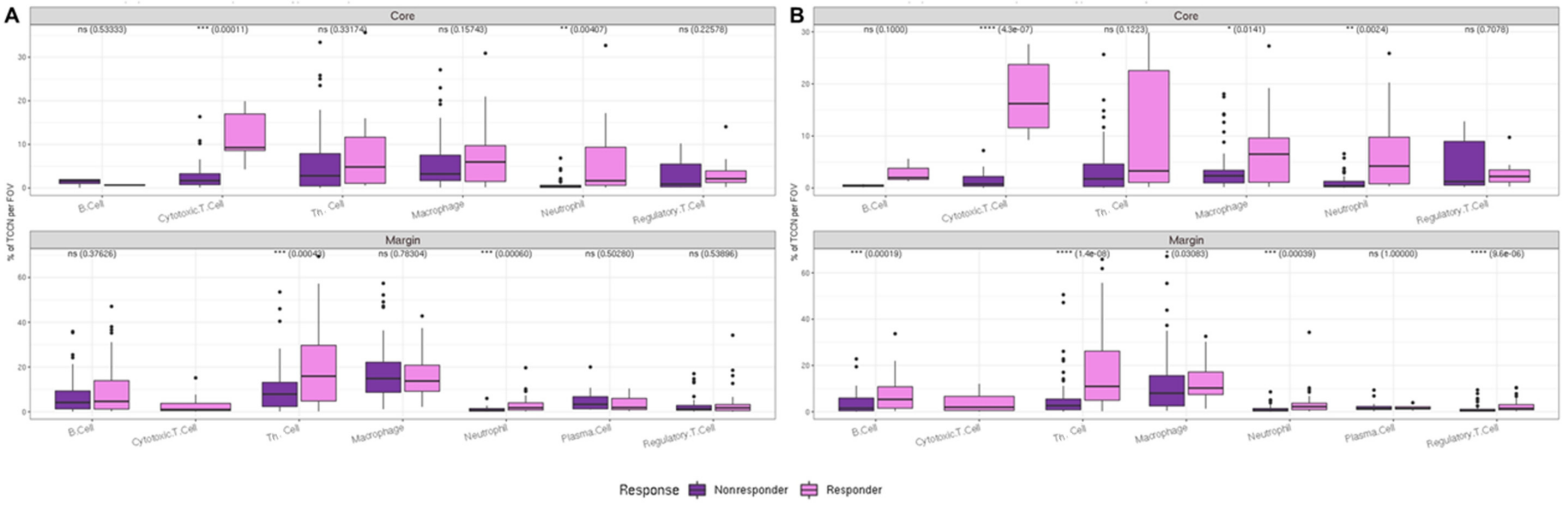
TCCNs by HLA-I status of the iTC. Box plots of immune cell subsets populating TCCNs with HLA-I^pos^ iTC (**A**) and HLA-I^neg^ iTC (**B**) in the tumor core and margin. Comparisons between responder and nonresponder TCCNs were compared using the Spearman correlation statistic.

Given the intrapatient heterogeneity of HLA-I tumor expression observed at the single-cell level, we expanded the composition and spatial analysis of TCCN to account for the HLA-I status of the index tumor cell (iTC; Fig. 4D). Generally, irrespective of anatomic region or HLA-I iTC status, responders had a greater percentage of TCCNs populated with immune cells compared with nonresponder but the immune composition of the TCCNs was distinct based on the anatomic region and HLA-I status of the iTC (Fig. 6). In the tumor core, responders displayed an increased prevalence for cytotoxic T cells and neutrophils in both HLA-I^pos^ and HLA-I^neg^ TCCNs while an increase of macrophages was only observed in HLA-I^neg^ iTC TCCNs of responders. In the tumor margin, Th cells and neutrophils more frequently populated responder TCCNs irrespective of HLA-I iTC status, while B cells, macrophages, and regulatory T cells were also increased in HLA-I^neg^ iTC TCCNs of responders. Taken together, these results suggest an increased utility for TCCN spatial analysis in further refining the immune composition profiles that distinguish the TIME of responders and nonresponders beyond single-cell analysis alone.

## Discussion

Deep profiling of the TIME is providing new insights into the clinical heterogeneity of lymph node metastases as it relates to immunotherapeutic response. Single cell–based technologies have revolutionized the granularity by which the composition of the TIME can be probed (18). With the advent of multiplex bioimaging platforms, the capacity to evaluate this single-cell composition of the TIME while simultaneously preserving the spatial landscape and inherent interactions between adjacent cells becomes possible (19). In this study, we applied a cyclic MxIF platform to construct the single-cell spatial profile and paracrine micro-niches surrounding individual tumor cells within the spatial landscape of the metastatic melanoma lymph node. Anatomic subregions within the lymph node demonstrated distinct composition profiles that coincided with clinical response to immunotherapy, namely increased cytotoxic T cells and macrophages in the tumor core of responders as previously demonstrated with demonstrated prognostic utility (33). Beyond single cells, the spatial preservation of the excisional lymph node tissue also enabled TIME evaluation at the level of TCCNs. In this setting, TCCNs demonstrated distinct immune signatures based on the anatomic region selected (core vs. margin) and the HLA-I functional status of the iTC.

Application of nearest neighborhood analysis to spatial mapping has been previously and extensively developed in the field of ecology, particularly in studies considering the spatial structures and diversity of forests (34–37). More recently, it has been adapted by numerous groups for multiplex imaging applications as a way to mathematically model single-cell data within a preserved spatial context (38, 39); however, the neighborhood size cutoff varies widely and often lacks biological rationale. In the current study, we derived the TCCN diameter from features of the cells that comprise the neighborhood of interest considering tumor subtype, nuclei size, and cellular composition within the anatomical subregion of a given FOV. We anticipate this derivation of a tissue and tumor-specific TCCN will provide a versatile, highly adaptable framework for future studies in different tumor types and tissue samples utilizing the spectrum of multiplex imaging modalities.

Multiplex imaging studies have predominantly focused on resolving clinical heterogeneity across a large patient cohort through analysis of tissue microarrays (22, 23). Building from these pioneering studies in spatial mapping, we chose to interrogate several FOVs within a single, spatially preserved lymph node biopsy sampling the distinct regions of the tumor core, margin, and lymphoid tissue. By sampling 323 FOVs in 14 metastatic lymph nodes, a more holistic analysis of the TIME composition as it applies to the tumor core, margin, and lymphoid tissue regions was able to be captured. While the single-cell composition identified known prognostic associations between cytotoxic T cells and clinical response to immunotherapy particularly in the tumor core, TCCN further resolved immune signatures to differentiate the TIME by clinical response. The increased prevalence of Th cells and neutrophils in margin-derived TCCNs of responders suggests these immune cell subsets at the tumor edge may mediate critical paracrine interactions in the context of immunotherapy. Further studies interrogating the spatial arrangement of these immune-enriched TCCNs across the TIME landscape may provide novel insights into the paracrine interactions that ultimately result in responsiveness to therapy.

Advanced, multiplexed bioimaging platforms are undoubtedly enhancing our understanding of the spatial TIME but are not without limitations. Classification remains a significant challenge for many multiplex bioimaging modalities. Despite a high level of classification assignment to each sample and corresponding FOV, it is plausible that cell types that are not quantified in the abundance plot for a given sample are underrepresented. To address this potential shortcoming, the monocyte cell class was not evaluated in the downstream TCCN analysis, except for MPO-expressing neutrophils (confirmed by serial H&E evaluation). In addition, further refinement of the antibody panel design in future studies, specifically the expansion of phenotypic markers and incorporation of functional markers including PDL1 will aid in refining classification for tumor and immune cell subsets. In our study, we selected patients with clear clinical response or failure to immunotherapy to consider two clinically distinct outcomes and the corresponding spatial landscapes. As such, our sample size was small and while insufficient to address sex-based differences, it was reflective of the clinical outcome experience as related to anti-PD1 immunotherapy treatment of stage IV melanoma (4–6). Future studies are needed to evaluate the potential utility of TCCN immune signatures as a prognostic feature in the pretreatment setting.

This study establishes MxIF as an effective platform to resolve patterns of paracrine interactions within the TIME of the metastatic melanoma lymph node as it relates to clinical response to PD1 immunotherapy. Through a comprehensive, multiplexed imaging approach, this work provides a framework for resolving spatial, cellular, and clinical heterogeneity in a biologically meaningful way utilizing the analytic unit of paracrine interaction, the TCCN. Our results demonstrate paracrine immune signatures within TCCNs are influenced by the anatomical region and HLA-I status of the iTC and refine the spatial landscape of the TIME beyond single-cell quantification alone. By demonstrating the viability of the MxIF INCell as a single-cell resolving biomaging platform, we have reaffirmed hypotheses surrounding the prognostic role of cytotoxic T cells and macrophages in the tumor core and identified new spatially relevant interactions between tumor and immune cell subsets with the potential to inform clinical responses to immunotherapy in the future.

## Supplementary Material

Supplementary Table 1 Antibody reagents and validation for MxIF panel design

Supplementary Table 2 Summary data of FOV distribution, single cells segmented, classified and clinical response to therapy

Supplementary Figure 1 Phenotypic Cell Classification Tree

Supplementary Figure 2 Virtual H&E images for whole excisional lymph node biopsies from each clinical case
