# Supplementary material for: Resolving the Heterogeneous Tumor-Centric Cellular Neighborhood through Multiplexed, Spatial Paracrine Interactions in the Setting of Immune Checkpoint Blockade
Source: Cancer Res Commun. 2022 Feb 10;2(2):78–89. doi: 10.1158/2767-9764.CRC-21-0146 (PMC9390837; doi:10.1158/2767-9764.CRC-21-0146)
Supplement: Supplementary Table 1 — Antibody reagents and validation for MxIF panel design [file crc-21-0146-s01.pdf]

Supplementary Table 1. Antibody Reagents and Validation for MxIF Panel Design

MxIF Antibodies for Panel Design							MxIF Antibody Validation	
**Marker Name**	**Manufacturer**	**Catalog Number**	**Clone**	**Conjugate**	**Positive Tissue**		**Routine Chromogenic IHC Reviewed**	**Serial H&E Reviewed**
CD20	abcam	ab166865	EP459Y	Cy2	Tonsil, Lymph node		X	X
CD8	Dako	M7103	C8/144B	Cy2	Tonsil, Lymph node		X	X
PD1	abcam	ab186928	EPR4877	Cy2	Tonsil, Lymph node		X	X
MART1	abcam	ab212362	M2-9E3	Cy2	Melanoma TMA		X	X
S100B	abcam	ab218956	S100B/1012	Cy2	Melanoma TMA		X	X
LYVE1	abcam	ab232935	EPR21857	Cy2	Lymph node			X
gp100	abcam	ab212829	HMB45	Cy2	Melanoma TMA		X	X
Ki67	abcam	ab231172	Sp6	Cy2	Tonsil, Lymph node, Melanoma TMA		X	X
CD10	Leica	NCL-CD10-270	56C6	Cy2	Tonsil, Lymph node			X
CD4	abcam	ab181724	EPR6855	Cy3	Tonsil, Lymph node		X	X
HLA-I	abcam	ab70328	EMR8-5	Cy3	Tonsil, Lymph node		X	X
CD163	Bio-Rad	MCA 1853	EDHu-1	Cy3	Tonsil, Lymph node		X	X
CD206	abcam	ab64693	poly	Cy3	Tonsil, Lymph node			X
CD56	abcam	ab9018	RNL-1	Cy3	Tonsil, Lymph node		X	X
CD31	Dako	M0823	JC70A	Cy3	Tonsil, Lymph node		X	X
CD27	abcam	ab192336	EPR8569	Cy3	Tonsil, Lymph node			X
CD38	abcam	ab204940	5C5C3	Cy3	Tonsil, Lymph node		X	X
CD45	abcam	ab10558	poly	Cy3	Tonsil, Lymph node		X	X
Kappa	abcam	ab134083	poly	Cy3	Tonsil, Lymph node			X
CD183	Sigma	SAB3501088	poly	Cy3	Tonsil, Lymph node			X
CD294	abcam	ab150632	poly	Cy3	Tonsil, Lymph node			X
MPO	abcam	ab221847	EPR20257	Cy3	Tonsil, Lymph node			X
NaKATPase	abcam	ab76020	EP1845Y	Cy5	Tonsil, Lymph node, Melanoma TMA			X
FoxP3	Biolegend	320102	206D	Cy5	Tonsil, Lymph node		X	X
CD3	Dako	M7254	F7.2.38	Cy5	Tonsil, Lymph node		X	X
CD14	abcam	ab214438	EPR3653	Cy5	Tonsil, Lymph node		X	X
HLA-II	invitrogen	MA1-80678	WR18	Cy5	Tonsil, Lymph node		X	X
Ribosomal protein S6	Santa Cruz	sc-74459	C-8	Cy5	Tonsil, Lymph node			X
CD68	Neomarkers	MS-397-P1ABX	KP1	Cy5	Tonsil, Lymph node		X	X
CD169	Santa Cruz	sc-53442	HSn 7D2	Cy5	Tonsil, Lymph node			X
β2m	abcam	ab181727	4G5A1	Cy5	Tonsil, Lymph node		X	X
CD16	Santa Cruz	sc-20052	DJ130c	Cy5	Tonsil, Lymph node			X
CD19	abcam	ab215382	EPR5906	Cy5	Tonsil, Lymph node			X
Lambda	abcam	ab195573	RM127	Cy5	Tonsil, Lymph node			X
cleaved Caspase 3	Cell Signaling	9664BF	D175	Cy5	Tonsil, Lymph node			X
