# Supplementary material for: Resolving the Heterogeneous Tumor-Centric Cellular Neighborhood through Multiplexed, Spatial Paracrine Interactions in the Setting of Immune Checkpoint Blockade
Source: Cancer Res Commun. 2022 Feb 10;2(2):78–89. doi: 10.1158/2767-9764.CRC-21-0146 (PMC9390837; doi:10.1158/2767-9764.CRC-21-0146)
Supplement: Supplementary Table 2 — Summary data of FOV distribution, single cells segmented, classified and clinical response to therapy [file crc-21-0146-s02.pdf]

Supplementary Table 2. Summary data of FOV distribution, single cells segmented, classified and clinical response to therapy (CR complete response; PD progressive disease)

Study ID	Total # FOVs	Core FOVs	Margin FOVs	Normal Adj FOVs	Segmented Cells	Classified Cells	Percent Classified	Clinical outcome
Mel4	20	12	8	0	85,914	84,216	98.0%	PD
Mel5	24	4	16	4	198,512	193,518	97.5%	PD
Mel10	20	5	10	5	119,006	111,171	93.4%	CR
Mel12	21	3	12	6	164,502	159,058	96.7%	CR
Mel14	30	9	15	6	181,065	177,132	97.8%	CR
Mel19	16	0	7	9	120,087	117,646	98.0%	CR
Mel21	36	19	16	1	171,233	167,102	97.6%	PD
Mel23	34	9	13	12	306,119	281,135	91.8%	PD
Mel25	18	9	9	0	53,689	51,922	96.7%	CR
Mel27	13	0	4	9	106,442	104,073	97.8%	CR
Mel28	19	8	8	3	92,061	89,241	96.9%	PD
Mel29	17	3	7	9	161,333	156,749	97.2%	PD
Mel30	18	4	11	3	113,808	107,098	94.1%	CR
Mel33	37	18	11	8	191,288	184,380	96.4%	PD
**Total**	**323**	***103***	***147***	***75***	**2,065,059**	**1,984,441**	**96.4%**	
