# Supplementary material for: Resolving the Heterogeneous Tumor-Centric Cellular Neighborhood through Multiplexed, Spatial Paracrine Interactions in the Setting of Immune Checkpoint Blockade
Source: Cancer Res Commun. 2022 Feb 10;2(2):78–89. doi: 10.1158/2767-9764.CRC-21-0146 (PMC9390837; doi:10.1158/2767-9764.CRC-21-0146)

Supplementary Figure 1. Phenotypic Cell Classification Decision Tree. Markers from panel design assigned to each cell phenotype for cell classification Figure created with Biorender.com

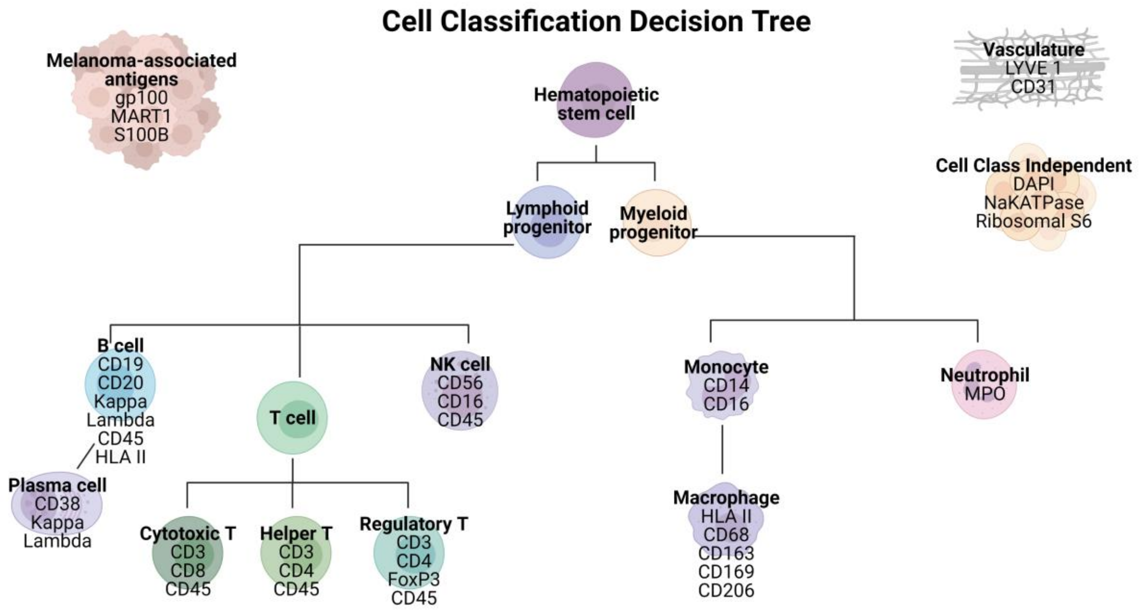

Supplement: Supplementary Figure 1 — Phenotypic Cell Classification Tree [file crc-21-0146-s03.pdf]
