# Supplementary material for: Resolving the Heterogeneous Tumor-Centric Cellular Neighborhood through Multiplexed, Spatial Paracrine Interactions in the Setting of Immune Checkpoint Blockade
Source: Cancer Res Commun. 2022 Feb 10;2(2):78–89. doi: 10.1158/2767-9764.CRC-21-0146 (PMC9390837; doi:10.1158/2767-9764.CRC-21-0146)

Supplementary Figure 2: virtual H&E images for whole excisional lymph node biopsies from each clinical case (n=14). Colored squares indicate pathologist-annotated FOVs selected for tumor core, margin and normal adjacent tissue.

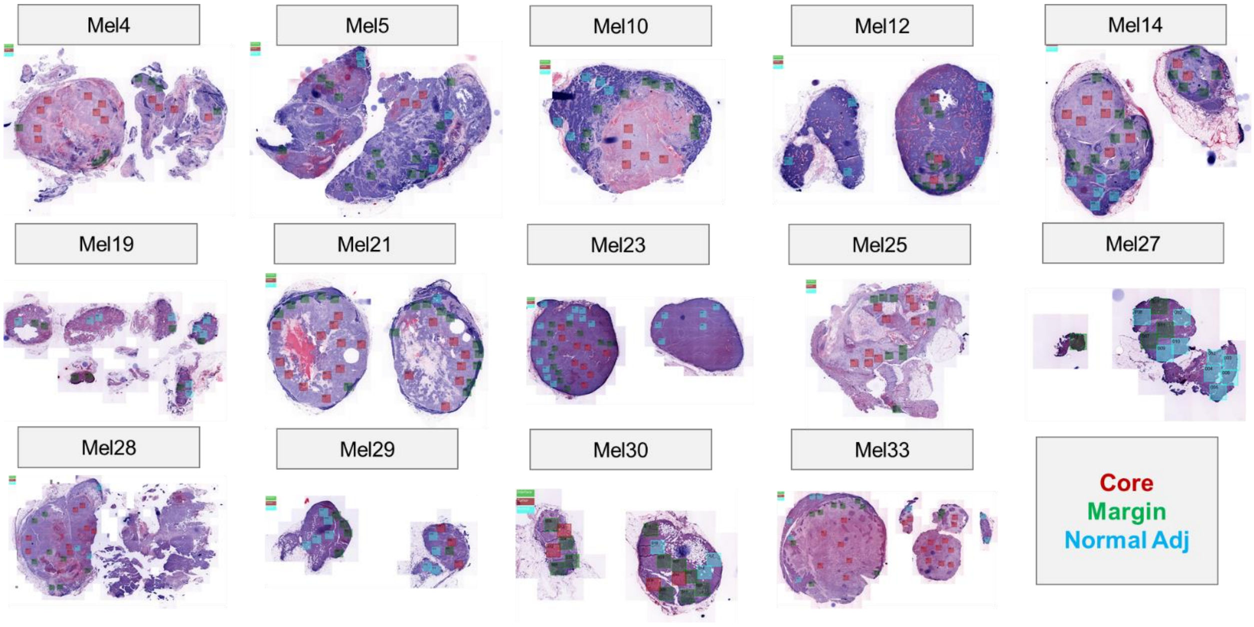

Supplement: Supplementary Figure 2 — Virtual H&E images for whole excisional lymph node biopsies from each clinical case [file crc-21-0146-s04.pdf]
